# Identification of FDA-approved Drugs Targeting Breast Cancer Stem Cells Along With Biomarkers of Sensitivity

**DOI:** 10.1038/srep02530

**Published:** 2013-08-28

**Authors:** Poornima Bhat-Nakshatri, Chirayu P. Goswami, Sunil Badve, George W. Sledge, Harikrishna Nakshatri

**Affiliations:** 1 Departments of Surgery, Indiana University School of Medicine, Indianapolis, IN 46202, USA; 2 Center for Computational Biology and Bioinformatics, Indiana University School of Medicine, Indianapolis, IN 46202, USA; 3 Pathology and Laboratory Medicine, Indiana University School of Medicine, Indianapolis, IN 46202, USA; 4 Medicine, Indiana University School of Medicine, Indianapolis, IN 46202, USA; 5 Biochemistry and Molecular Biology, Indiana University School of Medicine, Indianapolis, IN 46202, USA; 6 Current address: Stanford University School of Medicine, Paolo Alto, CA, USA.

## Abstract

Recently developed genomics-based tools are allowing repositioning of Food and Drug Administration (FDA)-approved drugs as cancer treatments, which were employed to identify drugs that target cancer stem cells (CSCs) of breast cancer. Gene expression datasets of CSCs from six studies were subjected to connectivity map to identify drugs that may ameliorate gene expression patterns unique to CSCs. All-trans retinoic acid (ATRA) was negatively connected with gene expression in CSCs. ATRA reduced mammosphere-forming ability of a subset of breast cancer cells, which correlated with induction of apoptosis, reduced expression of SOX2 but elevated expression of its antagonist CDX2. SOX2/CDX2 ratio had prognostic relevance in CSC-enriched breast cancers. K-ras mutant breast cancer cell line enriched for CSCs was resistant to ATRA, which was reversed by MAP kinase inhibitors. Thus, ATRA alone or in combination can be tested for efficacy using SOX2, CDX2, and K-ras mutation/MAPK activation status as biomarkers of response.

Cancer cell subpopulations with stem/progenitor cell-like properties have been described for several solid tumors^1^,^2^. These cancer cells termed cancer stem cells (CSCs) are isolated based on differential cell surface marker expression and then characterized for self-renewal and differentiation properties through in vitro sphere assays (mammospheres) and/or tumorigenicity in non-obese diabetic/severe combined immunodeficiency (NOD/SCID) mice^1^. At least two types of breast cancer cells display CSC properties: 1) CD44^+^/CD24^−^/Lineage^−^ cells, the first described CSCs, found mostly in basal-type breast cancers^3^; 2) Cancer cells that express higher levels of Aldehyde Dehydrogenase 1 (ALDH1+), which are present mostly in luminal breast cancers^4^. Additional markers that further refine CSCs including Delta-like (DLL), Delta/Notch-like EGF repeat containing (DNER), CD271, ganglioside GD2, and Dopamine receptors 3 and 5 have been reported^5^,^6^,^7^,^8^.

Although definition of CSCs remains largely operational, CSCs might explain tumor heterogeneity, chemotherapy/radiation resistance, and metastasis^1^. Endocrine- and chemotherapy-resistant luminal-type breast cancers acquire CSC properties with concomitant loss of luminal features and gain of basal-like features^9^,^10^. Neoadjuvant trials with docetaxel or letrozole (endocrine therapy) have shown enrichment of CSCs in residual luminal tumors^11^. Elevated levels of CSCs in primary tumors correlates with higher tumor grade, brain and lung relapse, and poor outcome^12^. A meta-dataset analysis involving seven independent breast cancer gene expression datasets has identified enrichment of four gene expression signatures including normal mammary stem cells and embryonic stem cell signatures in higher-grade tumors with CSC phenotype^12^.

Breast cancers are subclassified into five intrinsic subtypes^13^. Among these subtypes, claudin-low subtype is enriched for CSCs^14^. Claudin-low subtype breast cancers are triple negative breast cancers (TNBCs), which lack the expression of estrogen receptor (ER), progesterone receptor (PR), and HER2. Recent studies have further refined TNBCs into six subtypes based on gene expression patterns: basal-like 1 (BL-1), basal-like 2 (BL-2), mesenchymal (ML), mesenchymal-stem like (MSL), immunomodulatory (IM), and luminal androgen receptor (LAR)^15^. The gene expression pattern in MSL and ML subtypes overlaps with the gene expression pattern in CSCs and claudin-low subtype. Thus, three subtypes of breast cancers (claudin-low, MSL, and ML), high-grade breast cancers (G3), and tumors that are resistant to currently available therapies may require drugs that target CSCs.

Progress in developing drugs targeting CSCs has been slow. Salinomycin was recently suggested to preferentially target CD44+/CD24± CSCs in in vitro studies^16^. However, it is less likely to enter the clinic because it is equally toxic to normal stem cells in vivo^8^. IL-8/CXCR1/CXCR2 pathway is being considered to target CSCs^17^. However, for immediate need, repurposing of existing FDA approved drugs with additional considerations for biomarkers of drug sensitivity is the best option, which was investigated in this study.

## Results

### Connectivity map (CMAP) reveals the effect of ATRA in reversing CSC-enriched gene expression pattern

With recent advances in genomics, we now have tools to revisit reasons for failures of previous clinical trials and to identify biomarkers of drug sensitivity. We approached this issue by combining cancer stem cell genomics with connectivity map (CMAP)^18^,^19^. The CMAP is a database of gene expression profiles in four cell lines (MCF-7, HL-60, SKMEL5, and PC3) under treatment with differing concentrations of ~1000 FDA approved drugs. The database contains ~6100 gene expression profiles resulting from treatment of cell lines with different concentrations of these drugs^18^. The gene expression profiles from CMAP can be compared with gene expression profiles in other experiments to investigate how much expression in a condition correlate with expression resulting from drug treatment. The correlation is given a score from +1 (maximum positive correlation) to −1 (maximum negative correlation) based on the extent of correlation. Drugs that receive the score close to −1 are likely to have a therapeutic value since their gene expression profile is a reversal of profile present in the experimental condition. This approach has resulted in identification of Cimetidine, an antiulcer drug, as a potential therapy for lung cancer^19^. We performed CMAP analyses of gene expression datasets comparing MCF-10A CD44+/CD24− with CD44−/CD24+ subpopulation^20^, tumorigenic (CD44+/CD24−/Lin−) cells versus non-tumorigenic cells from primary tumors^21^, genes up or down-regulated in pooled normal and metastatic CD44+ breast cancer cells versus normal and metastatic CD24+ cells^22^,^23^, and transformed SSEA1+ CSC fibroblasts versus transformed SSEA1− fibroblasts^23^. Genes differentially expressed in CD271+ basal-like cells with CSC activity as well as in GD2-enriched cells, which overlap with CD44+/CD24− cells, were included^6^,^7^. Table 1 provides a partial list of highly connected drugs. All-trans retinoic acid (ATRA, also called Tretinoin) and the PPARγ agonist Poiglitazone (ACTOS) emerged as drug candidates that are negatively associated with CSC-enriched gene expression signatures. Since PPARγ agonists have been withdrawn from market but Tretinoin and drugs such as bexarotene (Targretin) with properties overlapping ATRA are in clinical use^24^,^25^, we evaluated the ability of ATRA in ameliorating CSC properties.

### ATRA is negatively connected with gene expression pattern in ML and MSL subtypes of TNBCs

Since MSL and ML subtypes of TNBCs are enriched for CD44+/CD24− cells^15^, we subjected gene expression signatures of subtypes of TNBCs to CMAP. This analysis allowed us to determine whether gene expression pattern in MSL and ML but not other subtypes of TNBCs negatively correlates with Tretinoin treatment (Table 2). Tretinoin (ATRA) had a score of −0.962 and −0.841 for MSL and ML subtypes, respectively. Interestingly, few drugs demonstrated opposing scores for different subtypes of TNBCs. For example, Trichostatin A score was +1 for LAR subtype, whereas it was −1 for IM subtype. Metformin, which is now being evaluated in breast cancer clinical trials, as well as paclitaxel, were ineffective against BL-1 and BL-2 subtypes with both scoring positive values. These observations, if true in vivo, highlight the need for biomarker driven clinical trial, which can exclude patients who may perform poorly under a specific therapeutic regime.

### ATRA induces differentiation of fulvestrant-resistant breast cancer cells with acquired CD44+/CD24+ phenotype

**Figure 1:**
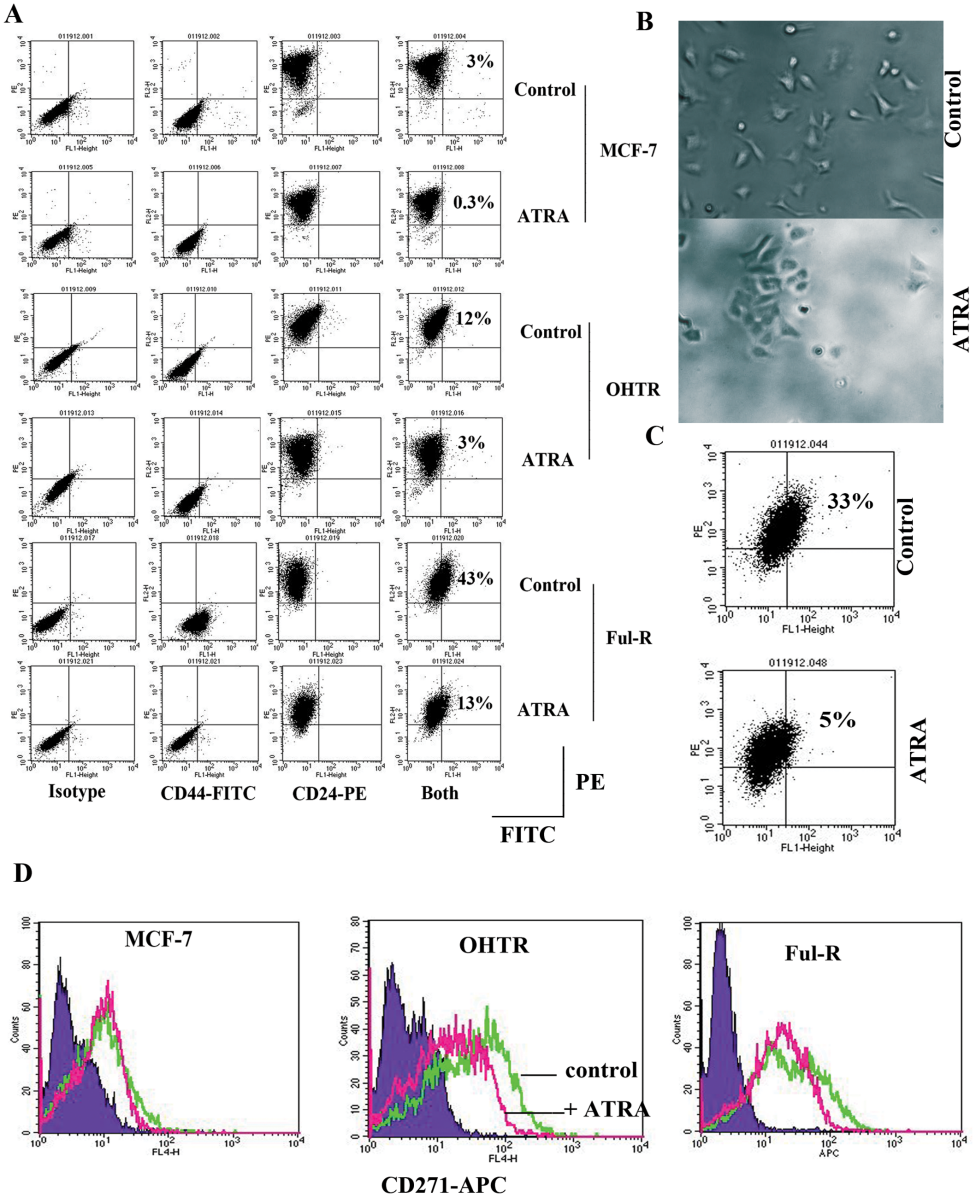
ATRA reverses CD44+/CD24+ phenotype of Ful-R cells. (A) Parental MCF-7, tamoxifen-resistant (OHTR), and Fulvestrant-resistant (Ful-R) variants were treated with ethanol or ATRA (1 μM) for 72 hours and subjected to flow cytometry as indicated. Percentage of CD44+/CD24+ Ful-R cells changed from 46 ± 3 to 13 ± 0.5 upon ATRA treatment (p = 0.003). (B) Morphological changes in Ful-R cells upon long-term exposure to ATRA. Cells were passaged for one month with or without ATRA treatment and photographed. (C) Cell surface CD44 and CD24 expression status of long-term ATRA treated Ful-R cells. (D) The effects of ATRA on CD271+ CSCs. MCF-7, OHTR, and Ful-R cells were treated with vehicle or ATRA (1 μM) for 72 hours and cell surface expression of CD271 was measured by flow cytometry. Representative isotype control (purple), untreated (green), and ATRA treated cell (pink) histograms from three experiments are shown.

CMAP results for breast cancer were derived from MCF-7 cells. These cells are estrogen receptor (ERα)-positive, proliferate in response to estradiol treatment, but are growth inhibited by anti-estrogens such as tamoxifen and fulvestrant. However, these cells, like in patients treated with anti-estrogens, eventually acquire resistance to treatment. To validate the results of CMAP analysis, we compared parental, 4-hydroxy tamoxifen-resistant (OHTR), and fulvestrant (Ful-R)-resistant cells for CD44 and CD24 status with or without ATRA treatment for 72 hours^26^. Note that OHTR and Ful-R cells were derived from a single cell MCF-7 clone and thus represent cells that have acquired resistance to drugs rather than clonal expansion of intrinsically drug resistant cells^26^. MCF-7 and OHTR cells were predominantly CD44−/CD24+ with ~3 and 12% being CD44+/CD24+ (Figure 1A). ATRA treatment reduced the levels of these double-positive cells. Ful-R cell line, which expresses very little ERα and displays epithelial to mesenchymal phenotype (EMT)^26^, contained equal number of CD44−/CD24+ and CD44+/CD24+ subpopulation. A recent study demonstrated enrichment of CD44−/CD24+ and CD44+/CD24+ cells in luminal and basal-A cell lines, respectively^27^. The above results thus suggest that fulvestrant resistance involves luminal cells acquiring basal-A phenotype. Additionally, we had previously demonstrated that MCF-7 cells that acquired CD44+/CD24+ phenotype display CSC phenotype^20^. Ful-R cells treated with ATRA demonstrated reversal to CD44−/CD24+ phenotype as percentage of cells with CD44+/CD24+ phenotype reduced from 46 ± 3% to 13 ± 0.5% (p < 0.01 untreated versus ATRA treated). The change in the CD44 expression status in Ful-R cells treated with ATRA is not likely due to direct effect of ATRA on CD44 transcription as CD44 gene lacks RAR binding sites.

To further ascertain the effect of ATRA in inducing differentiation of Ful-R cells, we maintained these cells in ATRA containing media for one month and then evaluated cell morphology, and CD44/CD24 expression status. Long-term ATRA treated cells reestablished cell-cell contact, lost some of the morphologic features of EMT including fibroblast-like appearance (Figure 1B) and CD44+/CD24+ phenotype (Figure 1C) suggesting reversal of EMT phenotype.

### OHTR resistance is associated with elevated CD271 positivity, which can be reversed by ATRA

To determine whether ATRA has any effect on CSC phenotype characterized using different markers, we examined MCF-7, OHTR, and Ful-R cells for CD271, which is a cell surface marker of a minority of basal-like cells with stem cell activity present in luminal cell lines such as MCF-7^6^. OHTR cells contained significantly higher CD271-positive cells (73 ± 9%) compared to MCF-7 (34 ± 10%, p = 0.009, OHTR versus MCF-7) or Ful-R cells (40 ± 16%, p = 0.04, OHTR versus Ful-R) (Figure 1D). Upon ATRA treatment, CD271-positive cells declined in all three-cell types, although maximum effects were observed in OHTR cells. Thus, ATRA reverses CSC phenotype based on two cell surface markers.

### ATRA reduces self-renewal as measured by mammosphere assay

**Figure 2:**
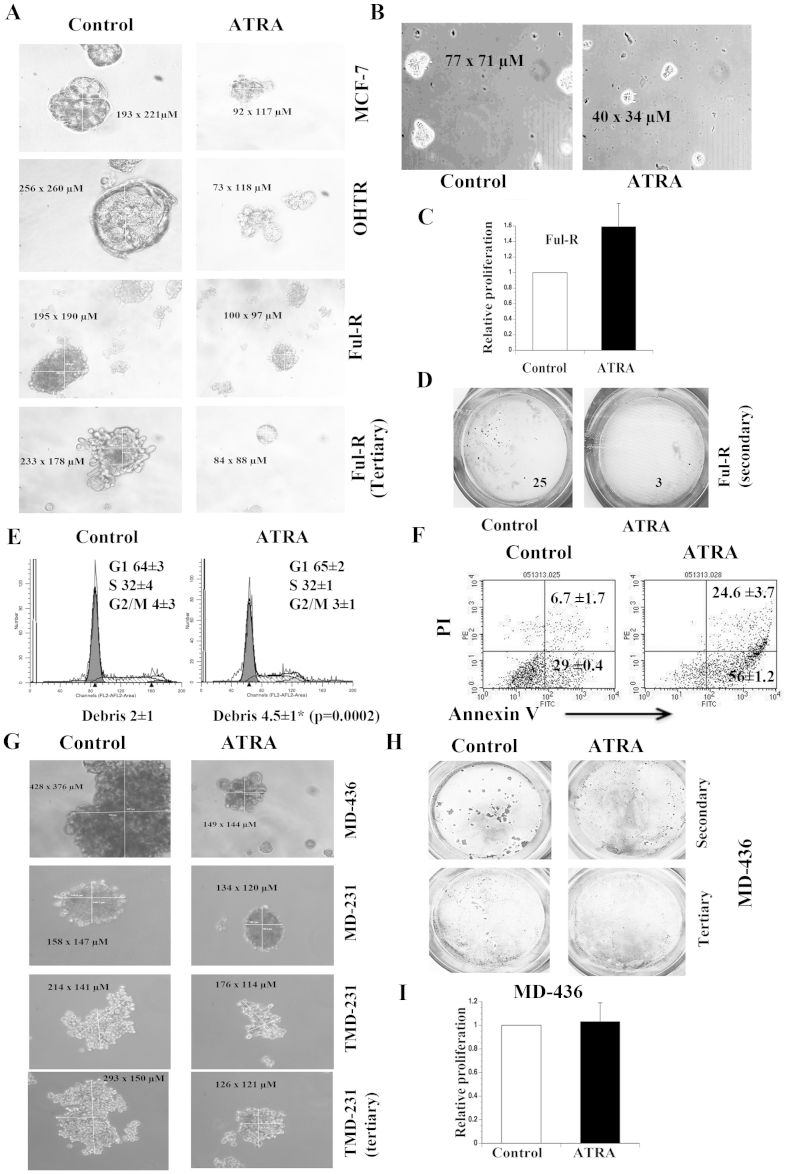
The effects of ATRA on mammospheres. (A) MCF-7, OHTR, and Ful-R cells were treated with ethanol or ATRA for 72 hours under adherent growth conditions and then subjected to mammosphere assay with or without ATRA. Mammospheres were photographed after 7 days. Size of mammospheres is indicated (n > 3). (B) Mammosphere formation in presence of methylcellulose. (C) The effect of ATRA on growth of Ful-R cells in 2D culture. Cells were treated with ATRA for 72 hours and BrdU-incorporation ELISA was used to measure cell proliferation. (D) Differences in secondary mammosphere formation by Ful-R cells with or without ATRA. Mammospheres were filtered through 40-micron filter and stained with Wright-Giemsa. (E) Cell cycle analysis of Ful-R cells-derived mammospheres grown with or without ATRA for 7 days. (F) Apoptotic cells in mammospheres with or without ATRA for 7 days (n = 3, Average ± SD, p < 0.002, untreated versus ATRA treated). (G) MDA-MB-231, TMD-231, and MDA-MB-436 cells-derived primary/secondary mammospheres with or without ATRA treatment. (H) Secondary and tertiary mammospheres from MDA-MB-436 cells were visualized using Wright-Giemsa stain. ATRA substantially reduced the size of secondary and tertiary mammospheres. (I) The effect of ATRA on proliferation of MDA-MB-436 cells in 2D culture. Cell proliferation was measured using BrDU-incorporation ELISA.

We next examined the effect of ATRA on CSC phenotype of MCF-7, OHTR, and Ful-R cells in mammosphere assay. Although cell aggregates were detected within 2–3 days of plating, the majority of these aggregates disappeared after few days, and mammospheres appeared after seven days. However, we do acknowledge the limitations of this assay, as it is often difficult to distinguish mammospheres from anoikis-resistant cell aggregates. ATRA reduced the size of primary mammospheres formed by MCF-7 and OHTR cells (Figure 2A). ATRA reduced the size of Ful-R cell-derived primary and tertiary mammospheres (Figure 2A). To minimize cell aggregation, we repeated mammosphere assay in media containing 1% methylcellulose, which increased media viscosity. ATRA reduced the size of mammospheres formed under this growth condition (Figure 2B). It is believed that the size of neurosphere reflects self-renewal rate^28^,^29^. Thus, the observed effect of ATRA on the size of the mammospheres may indicate its ability to slow the self-renewal process. Interestingly, ATRA had a modest growth stimulatory effect on Ful-R cells under 2D growth conditions (Figure 2C). Thus, appropriate growth conditions are required to assess the effects of ATRA and ATRA-resistant phenotype evident in 2D culture may not be manifested under 3D or stem cell culture conditions.

Quantitating mammospheres is often difficult. We devised a new strategy for visualizing mammospheres by filtering through 40-micron filter to remove single cell and small aggregates. The mammospheres on the top of the filter were fixed and stained with Wright-Giemsa. ATRA treated Ful-R cells showed significantly lower number of secondary and tertiary mammospheres compared to control cells (Figure 2D and data not shown).

We next performed cell cycle analysis of Ful-R cells grown under mammosphere condition with or without ATRA treatment. Untreated and ATRA-treated mammospheres showed typical dividing cell cycle pattern (Figure 2E). However, ATRA-treated mammospheres had higher percentage of debris indicating elevated cell death. We measured apoptosis in mammospheres under untreated and ATRA treated condition using Annexin V and flow cytometry. Mammospheres were trypsinized and single cells were stained for Annexin V and propidium iodide. ATRA treated mammospheres demonstrated ~3-fold increase in apoptosis/necroptosis compared to untreated mammospheres (Figure 2F). These results indicate a pro-apoptotic function of ATRA, which is not usually observed under 2D growth conditions.

### Cell type specificity in ATRA action on MSL subtype of TNBCs

To further demonstrate an effect of ATRA on CSC phenotype, we subjected MDA-MB-231 and MDA-MB-436 cells, both representing MSL subtype of TNBCs^15^, to mammosphere assay with or without ATRA treatment. The size of mammosphere was significantly reduced in MDA-MB-436 cells treated with ATRA compared to untreated controls (Figure 2G). Although ATRA reduced the size of mammospheres in MDA-MB-231 and TMD-231 cells, the effect was modest in both primary and tertiary mammospheres (Figure 2G). ATRA-treated MDA-MB-436 cells formed considerably lower number of secondary and tertiary mammospheres, although it had no effect on proliferation of cells under 2D culture (Figure 2H, I). These results suggest that ATRA has cell type specific effects in reversing CSC properties based on mammosphere assay.

### ATRA-mediated reduction in CSC properties correlates with repression of select CSC-associated genes

**Figure 3:**
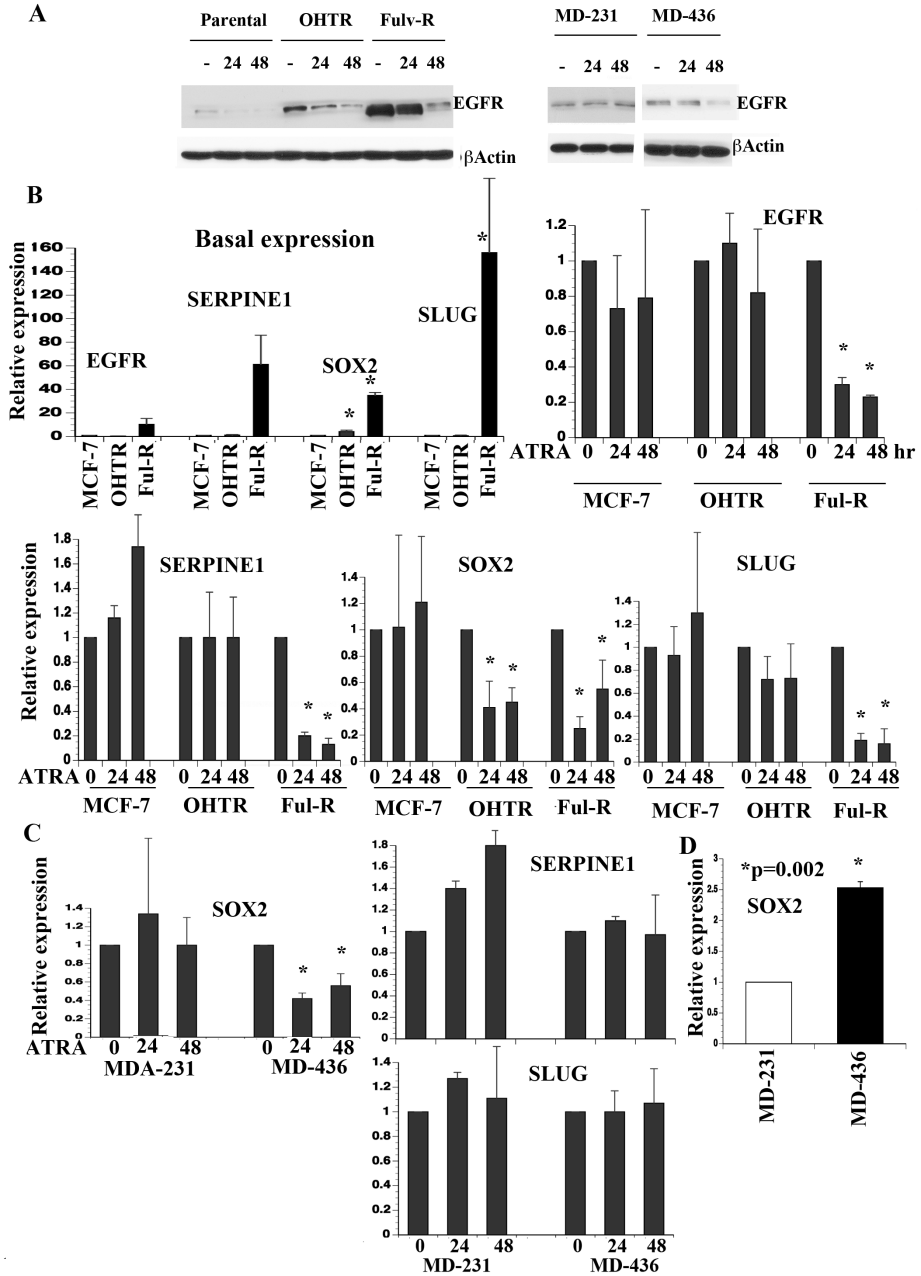
The effects of ATRA on the expression of CSC-associated genes. (A) ATRA reduced EGFR protein in all but MDA-MB-231 cells. Experiments were conducted in identical condition and cropped blots are shown. Full-length gels are shown in supplementary Figure 1 (Figure S1). (B) The effect of ATRA on mRNA levels of EGFR, SERPINE1, SOX2, and SLUG in parental MCF-7, OHTR, and Ful-R cells. Differences in the basal expression of these genes amongst three cell types are shown (top left). The effect of ATRA on gene expression is presented after normalizing basal expression to one in each cell type. Cells were treated with ATRA (1 μM) for indicated time and mRNA levels were measured by qRT-PCR (n = 3). *p < 0.001, untreated versus ATRA treated. (C) The effects of ATRA on SERPINE1, SOX2, and SLUG expression in MDA-MB-231 and MDA-MB-436 cells. ATRA reduced SOX2 expression but not other genes in MDA-MB-436 cells. *p < 0.01 untreated versus treated. (D) MDA-MB-231 cells express lower levels of SOX2 compared to MDA-MB-436 cells.

Several reports have shown induction of CSC-like phenotype upon EMT and non-CSCs acquiring CSC properties upon overexpression of EMT-associated genes such as SNAI2/SLUG^20^,^30^,^31^. Another recent study demonstrated a role of embryonic lineage commitment gene SOX2 in inducing CSCs and both SOX2 and SLUG are enriched in the recently described CD271-positive basal-like CSCs^6^,^32^. To identify potential targets of ATRA in cancer cells that have acquired CSC properties through EMT process, we performed EMT array analysis of untreated and ATRA treated Ful-R cells. These preliminary analyses indicated ATRA repressing AHNAK, CAV2, CDH2, EGFR, FGFBP1, IGFBP4, JAG1, MMP9, Notch1, SERPINE1, SNAI2/SLUG, and TGFβ1 (data not shown). None of these genes was repressed by ATRA in partially responsive MDA-MB-231 cells (data not shown). EGFR is of specific interest as the tumor-specific constitutively active variant III is associated with CD44+/CD24− subpopulation of breast CSCs^33^. Consistent with this possibility, EGFR showed cell type specific differences in expression as well as protein species. MCF-7, OHTR, and Ful-R cells expressed EGFR of different mobility with Ful-R cells expressing a low molecular weight EGFR (Figure 3A). EGFR protein levels were higher in OHTR and Ful-R cells compared to parental cells (Figure 3A). Irrespective of levels and isoforms, ATRA reduced the levels of EGFR in all three-cell types (Figure 3A).

The expression levels of EGFR, SERPINE1, SOX2, and SLUG in multiple cell types were examined by qRT-PCR. Basal expression of these genes was higher in Ful-R cells compared to MCF-7 or OHTR cells although statistical significance was achieved only with SOX2 and SLUG due to experimental variability (Figure 3B). OHTR and Ful-R cells expressed higher levels of SOX2 compared to MCF-7 (p < 0.004, Figure 3B, top left). ATRA reduced EGFR, SERPINE1, SOX2, and SLUG in Ful-R cells but only SOX2 in OHTR cells (Figure 3B, bottom center and right). Because of very low basal expression, the effect of ATRA on SOX2 expression in MCF-7 cells could not be reliably measured.

To further extend the correlation between ATRA-mediated reduction in CSC properties and loss of SOX2 expression, we measured the effect of ATRA on SOX2 expression in MDA-MB-231 and MDA-MB-436 cells. ATRA repressed SOX2 expression in only MDA-MB-436 cells (Figure 3C). It appears that MDA-MB-231 cells are not dependent on SOX2 to maintain CSC phenotype because the basal expression of this gene was 2.5 fold lower compared to MDA-MB-436 cells (Figure 3D). Thus, ATRA-mediated effects on CSCs consistently correlated with repression of SOX2 and EGFR.

**Figure 4:**
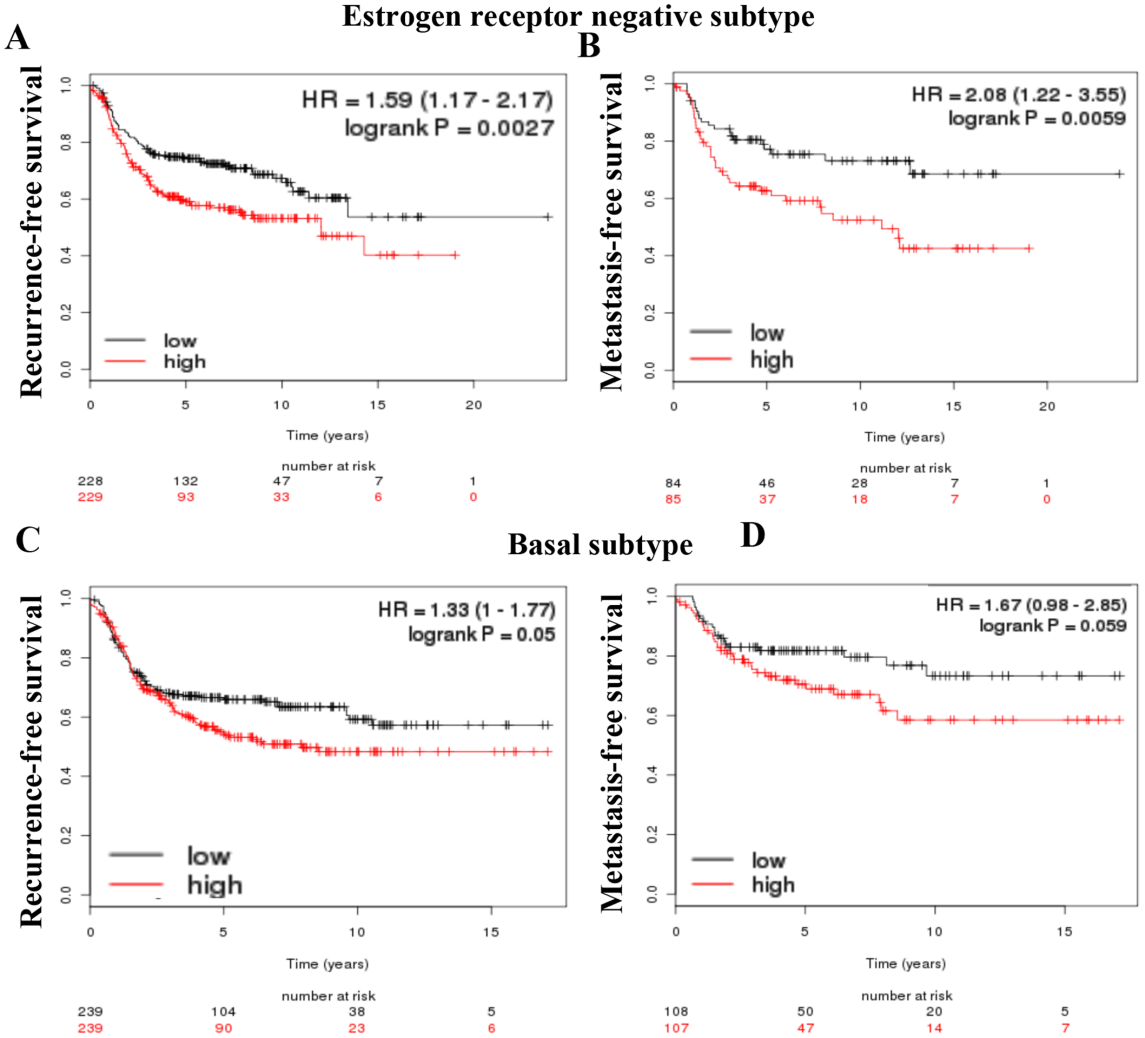
Prognostic relevance of combined expression of ATRA-repressible genes EGFR, SERPINE1, SLUG, and SOX2. Recurrence-free and metastasis-free survival of patients with ER-negative (A and B) or basal type (C and D) breast cancer expressing high (red) or low (black) levels of EGFR, SERPINE1, SLUG and SOX2.

We next examined whether EGFR, SERPINE1, SLUG, and SOX2, which are repressed by ATRA in a cell type dependent manner, constitute a prognostic signature in breast cancer using the publicly available database^34^. Elevated combined expression levels of these four genes were associated poor recurrence-free and distant metastasis-free survival outcome in patients with ER-negative breast cancer (Figure 4A–B). Analysis of different subtypes of breast cancer showed prognostic relevance of these genes in basal subtype but not luminal A, luminal B or HER2 subtypes (Figure 4C–D and data not shown). SOX2 plus EGFR had similar prognostic impact on ER-negative but not specific subtypes of breast cancer (data not shown). Collectively, these results illustrate that CSC-associated genes that are targets of ATRA have prognostic implications in breast cancer.

### ATRA induces the expression of SOX2 antagonist CDX2

**Figure 5:**
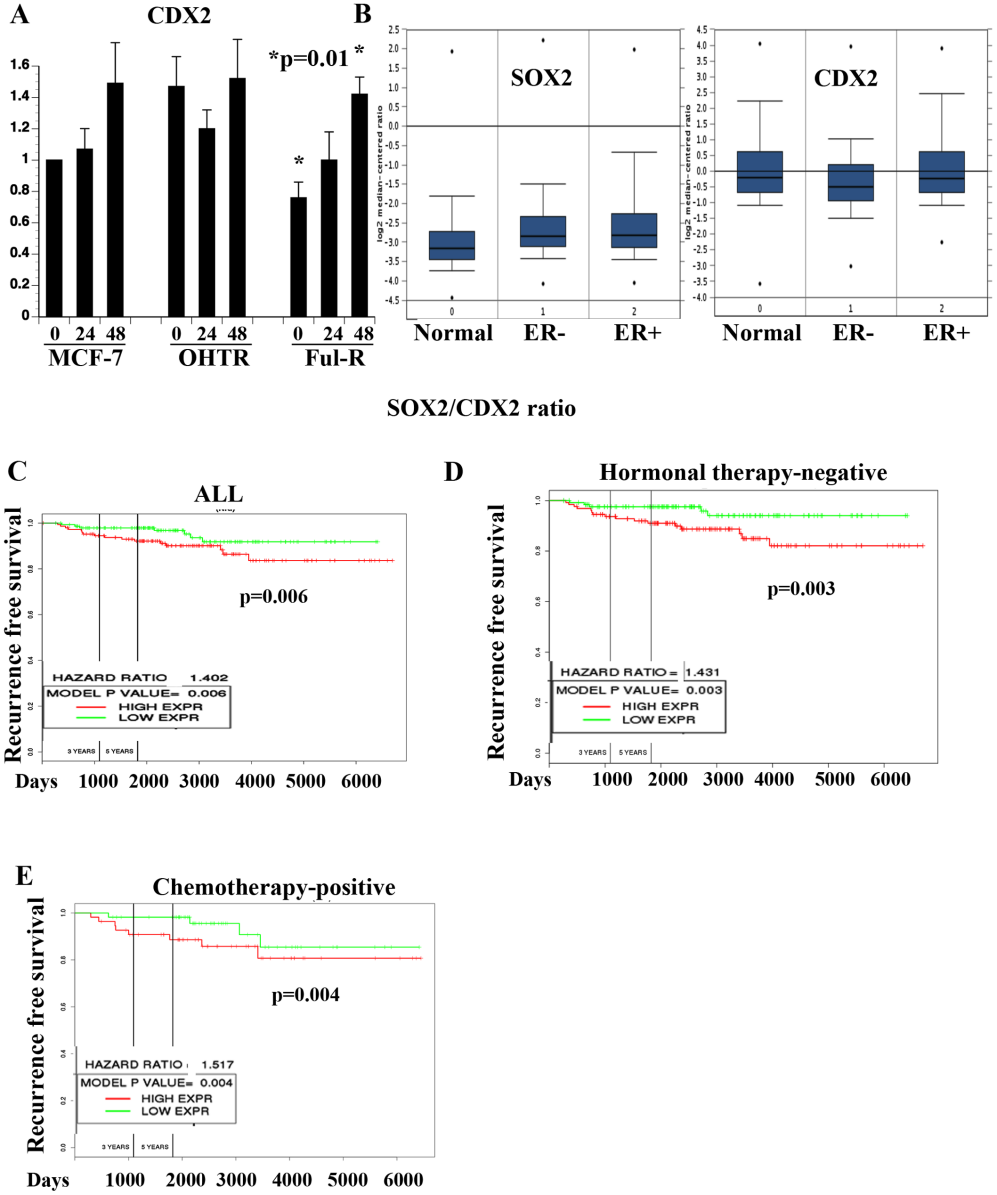
SOX2/CDX2 ratio has prognostic implications. (A) ATRA induced CDX2 expression in Ful-R cells. qRT-PCR was used to measure CDX2 levels. (B) SOX2 and CDX2 mRNA levels in adjoining normal breast, ER-positive and ER-negative breast cancer. Oncomine database was used to analyze the TCGA dataset. (C–E) Prognostic relevance of SOX2/CDX2 ratio in breast cancer. NKI dataset was analyzed for SOX2/CDX2 ratio.

Agonistic and antagonistic interactions between cell type specific transcription regulators and SOX2 are required for cell fate determination during development and to maintain homeostasis in adult tissues^35^. We first surveyed these SOX2-associated transcription factors (Oct4, Tbx6, Pax6, MITF, Nkx2.1, and Cdx2) for prognostic relevance in breast cancer using public database^34^, their relationship to ATRA signaling, and direct regulation by SOX2. This preliminary screening suggested CDX2 as a SOX2 antagonist that is likely to be under the control of ATRA. SOX2 has previously been shown to repress CDX2, whereas CDX2 represses SOX2^36^,^37^. ATRA increased CDX2 expression in Ful-R cells (Figure 5A). ATRA had no effect on CDX2 expression in MDA-MB-231 and MDA-MB-436 cells (data not shown). These results show ATRA-mediated loss of CSC phenotype in certain cell types is associated with altered SOX2:CDX2 ratio.

### SOX2/CDX2 ratio has prognostic implication in breast cancer

CDX2 has not been studied extensively in breast cancer. However, previous studies have shown a good prognostic value of CDX2 in gastric, ovarian, and pancreatic cancer^38^,^39^,^40^. Oncomine analysis of TCGA data^41^ revealed similar CDX2 expression levels between adjacent normal breast and ER-positive breast cancer but reduced expression in ER-negative breast cancer (Figure 5B). In contrast, SOX2 expression was higher in both ER-positive and ER-negative breast cancer compared to normal adjacent tissue (Figure 5B). The concept map analysis using Oncomine revealed that CDX2 as one of the top 1% of the underexpressed gene in all but one breast cancer datasets. Similar analysis of SOX2 did not show any specific pattern (data not shown). Since SOX2 and CDX2 have been shown to repress each other's expression, we next asked whether ratio between two genes has an impact on breast cancer outcome. NKI dataset is the most widely used dataset of this type of studies^42^. Higher SOX2/CDX2 ratio was associated with poor recurrence-free survival (Figure 5C). In addition, elevated SOX2/CDX2 ratio correlated with worst outcome in patients who did not receive hormonal therapy (mostly ER-negative) or received chemotherapy (Figure D, E). Similar results were obtained using a different dataset (data not shown)^43^. These results clearly demonstrate relevance of SOX2 and CDX2 ratio in breast cancer outcome. Our results show the ability of ATRA to reverse this ratio in a cell type-dependent manner. However, our attempts to knockdown SOX2 and CDX2 expression to significant levels in Ful-R cells using siRNA were not successful. Therefore, functional role of these genes in mediating ATRA effects on mammosphere needs to be verified through further experimentation.

### PD0325901 (Selumetinib), a MEK inhibitor, sensitizes MDA-MB-231 cells to ATRA

**Figure 6:**
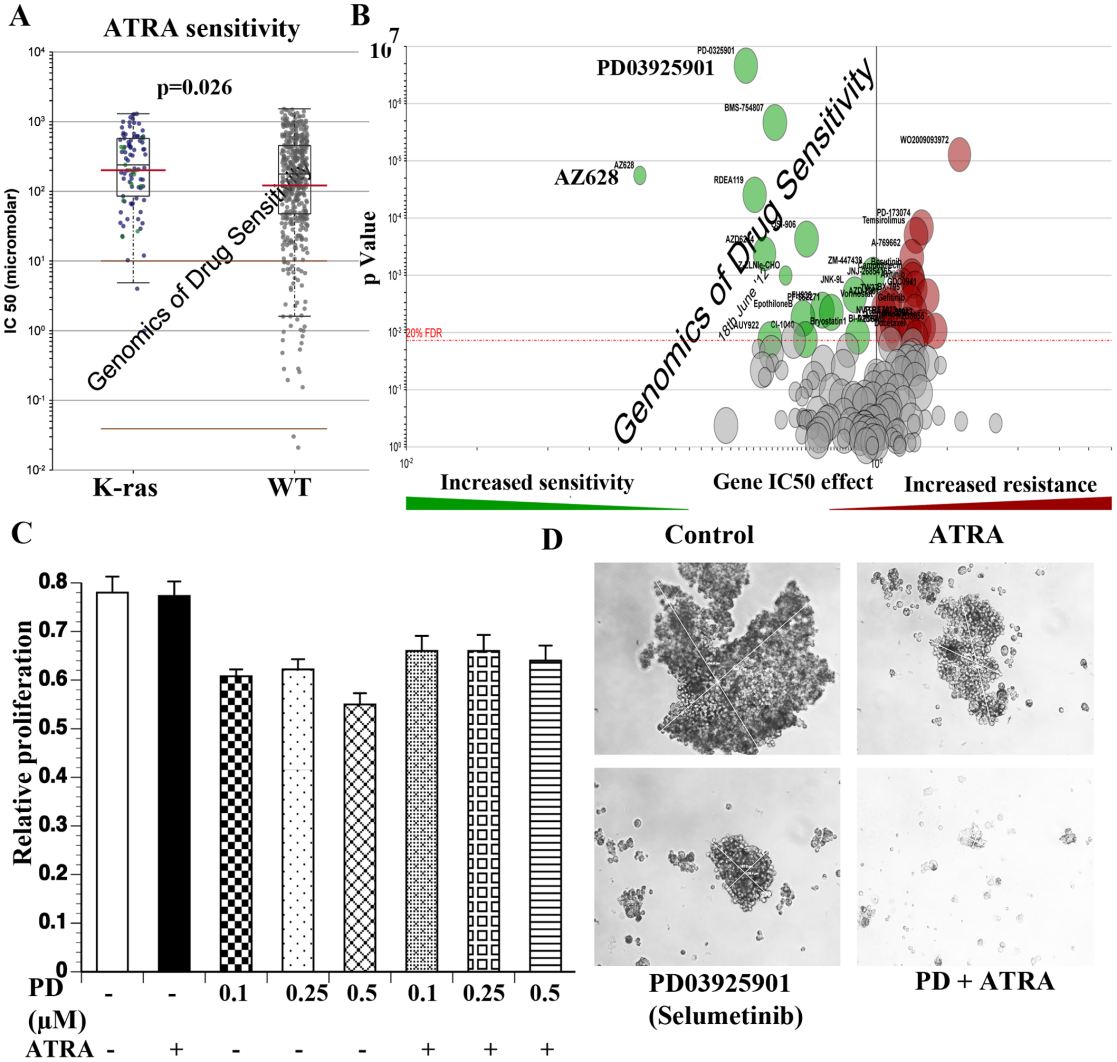
PD03925901 (Selumetinib) increased sensitivity of MDA-MB-231 cells to ATRA. (A) K-ras mutation confers resistance to ATRA. ATRA IC50 values for K-ras mutant and wild type cell lines are indicated. (B) Drug sensitivity of K-ras mutant cell lines. K-ras mutant cell lines are sensitive to drugs shown in green, which include AZ628 and PD03925901. These K-ras mutant cell lines are resistant to several other drugs, which are shown in red. (C) PD03925901 with or without ATRA has minimum effect on proliferation of MDA-MB-231 cells in 2D culture. (D) PD03925901 (0.5 μM) with ATRA (1 μM) inhibited mammosphere formation.

Despite displaying CD44+/CD24− features and characteristics of MSL subtype of TNBC, which scored negatively with ATRA in CMAP, MDA-MB-231 cells were resistant to ATRA. To determine the potential mechanisms of ATRA resistance of these cells, we analyzed COSMIC (Catalogue of Somatic Mutations in Cancer) database of Sanger Institute for cancer-associated mutations that confer resistance to ATRA. Among nine cancer-associated mutations that determine sensitivity to ATRA (Notch 1, BCR_ABL, KIT, FLT3, APC, TET2, K-ras, ALK, MLL_AFF1), KRAS, APC, and KIT mutations are associated with resistance to ATRA (Figure 6A, only K-ras is shown). MDA-MB-231 cells carry K-ras mutation (G13D) raising the possibility that this mutation contributes to ATRA resistance^44^. We used the same database to determine drugs that are effective against cell lines with K-ras mutation. PD0325901, a MEK inhibitor, and AZ628, a C-RAF inhibitor, were effective against cell lines with K-ras mutation (Figure 6B). Since PD0325901 (Selumetinib) is already in clinic, we examined its ability to inhibit proliferation of MDA-MB-231 in 2D culture and to form mammospheres with or without ATRA treatment. PD0325901 had minimum effect on MDA-MB-231 proliferation in 2D cultures with or without ATRA treatment (Figure 6C) but substantially reduced mammosphere formation when combined with ATRA (Figure 6D). These results suggest that genomic make up of cancer cells can be utilized to develop combination therapies involving pathway-specific drugs and ATRA.

## Discussion

Despite controversies surrounding the cancer stem cell hypothesis, patients with tumors that are enriched for gene expression signatures of CSCs encounter rapid disease progression and poor outcome^12^. EMT is one of the mechanisms by which cancer cells acquire CSC properties^20^,^30^. More often, residual tumors after therapy express higher levels of EMT-inducing genes, display elevated mammosphere forming ability, and have elevated TGFβ-signature score^11^,^45^. At present, there are no drugs in clinical use that target CSCs. Considering this need, we adapted CMAP approach. ATRA identified in this screen is more effective in reducing CSC phenotype and CSC-associated gene expression in cancer cells that have acquired CSC phenotype during the course of developing resistance to a targeted therapy. Ful-R cells, a fulvestrant resistant cell line derived from a single cell clone of MCF-7, were more sensitive to ATRA than MDA-MB-436 and MDA-MB-231 cells, which most likely represent intrinsic CSCs^31^.

ATRA, which came into solid tumor oncology clinic after extensive preclinical studies, performed very poorly in clinical trials. A recent clinical trial of ATRA in combination with taxol in patients with recurrent or metastatic disease revealed overall clinical benefit of 76.4% with a relatively high rates of stable disease^46^. However, these studies were not biomarker driven. Our results demonstrating ATRA being effective against tumor cells that have acquired CSC phenotype suggests its utility in a specific group of patients who has failed hormonal therapy. Recent neoadjuvant trials with hormonal therapy have shown enrichment of cancer cells with CSC properties in residual tumors^11^. Whether combining ATRA with anti-hormonal therapy will be an effective strategy to eliminate CSCs remains to be determined.

ATRA reduced mammosphere-forming ability of cell lines that expressed higher levels of SOX2 suggesting that only the cancer cells that are dependent on SOX2 for self-renewal are responsive to ATRA. ATRA also reduced the levels of EGFR, SERPINE1, and SLUG in a cell type-dependent manner. Both SERPINE1 and SLUG overexpression is linked to higher tumor grade and poor outcome in breast cancer patients^12^. Interestingly, in hepatocellular carcinoma, SOX2 induces the expression of SLUG^47^. SLUG is the main suppressor of human breast progenitor cell lineage commitment and differentiation and is aberrantly expressed in BRCA1 mutant tissue^48^. EGFR and SOX2 constitute a feedback loop that positively regulates self-renewal of neural stem cells^49^. The ability of ATRA to inhibit EGFR and SOX2 in all responsive cell lines, and SERPINE1 and SLUG in few cell types and repression of these genes correlating with diminished CSC phenotype raise the possibility of using these genes as biomarkers to distinguish ATRA responders from non-responders. CDX2, an antagonist of SOX2, can also be included as a biomarker as it is ATRA-inducible in responsive cell line. Consistent with this possibility, our analysis of gene expression array datasets revealed the prognostic relevance of these ATRA targets (Figure 4 and 5).

Recent studies in pancreatic cancer have identified biomarkers of ATRA response^50^. Two intracellular ATRA binding proteins, FABP5 and CRABPII, determine whether ATRA activates RAR:RXR signaling or RXR:PPARβ/δ signaling^51^,^52^. FABP5 directs ATRA to RXR/PPARβ/δ signaling, whereas CRABPII directs ATRA towards RAR:RXR signaling. Therefore, pancreatic cancer with lower levels of FABP5 retain sensitivity to ATRA compared to cells with high level of this intracellular retinoid binding protein^50^. An inverse correlation was observed with CRABPII. If FABP5 and CRABPII function similarly in breast tissues, CDX2, EGFR, SERPNIE1, SLUG, SOX2, FABP5, and CRABPII may serve as biomarkers of ATRA response. Additional preclinical studies are required to test this possibility.

MDA-MB-231 cells, which are CD44+/CD24− but appear SOX2-independent, were least sensitive to ATRA. However, this cell line has K-ras mutation, which is known to confer resistance to ATRA^53^. Inhibition of MEK, the downstream target of K-ras, restored sensitivity to ATRA in mammosphere assay. Thus, K-ras mutation and MEK activation status are additional biomarkers of ATRA sensitivity.

ATRA works through nuclear receptors, which are expressed at variable levels in different subtypes of breast cancer including cancers enriched for CSCs. Cell lines used in this study express all three RARs and RXRs based on the analysis of mRNA levels available publicly^54^. Similar to our study, Ginestier et al showed the ability of ATRA to inhibit mammosphere formation by ALDH1-positive CSCs^55^. Papi et al recently demonstrated the effect of ATRA and the RXR-specific ligand 6-OH-11-O-hydroxyphenanthrene in reducing CSC phenotype of breast cancer cells by targeting NF-κB pathway^56^. Our study clearly shows not all cancer cell lines with CSC phenotype are responsive to ATRA suggesting the existence additional determinants of ATRA action on CSCs. As ATRA repressed SOX2 in only responsive cell lines, understanding how ATRA represses SOX2 may provide insights into mechanisms and barriers involved in ATRA action. Collectively, our results should stimulate interest in developing ATRA based therapy for specific subtypes of breast cancer, which additionally considers biomarker driven patient selection and cancer genome-based combination therapies.

## Methods

### Cell lines

A single cell derived MCF-7 clone expressing ERE-Luciferase (parental), 4-hydoxy tamoxifen-resistant variant (OHTR) and fulvestrant-resistant variant (Ful-R) cell lines have been described previously and are generous gift from Dr. Ken Nephew^26^. MDA-MB-231, TMD-231, and MDA-MB-436 cells have been described previously^31^. Most of the studies with MDA-MB-231 cell line were done using a variant selected from a mammary fat pad tumor in nude mice (TMD-231). All cell lines were maintained in phenol red-free MEM with five percent dextran-charcoal treated fetal calf serum for at least four days prior to starting experiments and all experiments except mammosphere assays were done in this medium.

### Mammosphere assay

5,000 to 20,000 cells were plated on ultra-low adherent six well or 100 mm plates in MammoCult media from Stemcell Technologies (Vancouver, BC, Canada) as per instruction from manufacturers. Although few clumps with 10–20 cells were observed a day or two after plating, these clumps disappeared and mammospheres were detected by seven days. Mammospheres were visualized and photographed. For secondary and tertiary cultures, mammospheres were collected by centrifugation, washed, trypsinized, and equal number of cells were replated in mammosphere media. At the end of the experiment, mammospheres were filtered through 40-microfilter. Filters were stained with Wright-Giemsa stain (Fisher Diagnostics, VA, USA) and mammospheres were counted.

### RNA extraction and quantitative reverse transcription polymerase chain reaction (qRT-PCR)

RNA was isolated using the RNAeasy kit from Qiagen (Valencia, CA, USA). Single strand cDNA was synthesized using single strand synthesis kit (Bio-Rad Laboratories, Hercules, CA). qPCR was performed in duplicate measurements using syber-green PCR mix (Applied Biosystems) and specific primers on the qPCR instrument (Applied Biosystems). β-actin served as a normalization control. Sequences of primers used are provided in supplementary Table 1. EMT array analysis was done using PAHS-090 array from SA Biosciences (Valencia, CA, USA) as per instructions from the manufacturer.

### Analysis of publicly available databases

Oncomine database was used to determine the expression pattern of SOX2 and CDX2 in TCGA dataset^41^, whereas in-house database was used to determine the prognostic importance of SOX2/CDX2 ratio in NKI dataset^42^. Gene array datasets used for connectivity map are described in the text. The prognostic value of combined EGFR, SLUG/SNAI2, SERPINE1, and SOX2 was determined using KMplot^34^.

### Cell extracts and western bot analysis

Whole cell lysates were prepared in RIPA buffer and western blotting was preformed as described previously^20^. Antibody against EGFR was purchased from Santa Cruz biotechnology (Santa Cruz, CA, USA).

### Cell cycle, apoptosis, and flow cytometry

Flow cytometry was performed as described previously^31^. CD44-FITC and CD24-PE antibodies were purchased from BD Biosciences (San Jose, CA, USA), whereas CD271-APC antibody was from Biolegend (San Diego, CA, USA). Cell cycle analysis was performed as described previously^57^. Apoptosis assay of mammospheres was performed using Annexin V apoptosis assay kit as recommended by manufacturers (Invitrogen, Carlsbad, CA, USA). Representative data from three or more experiments are presented.

### Statistical analysis

qRT-PCR, flow cytometry and proliferation assay results were subjected unpaired *t* test using GraphPad Prism software (Graphpad.com). The two-tailed *p* values of <0.05 were considered statistically significant.

## Author Contributions

P.N.: Conception and design, collection and assembly of data; C.G.: Data analysis; S.B.: Conception and design; G.S.: Conception and design; H.N.: Conception and design, manuscript writing, and final approval of manuscript.

## Supplementary Material

Supplementary Information Supplementary Table S1 and Figure S1

## Figures and Tables

**Table 1:** Connectivity map analysis of CSCs versus non-CSCs. Negatively connected drugs are in bold. Rank corresponds to order of drugs giving score +1 to −1 and ranges from 1 to 6100. Drug at 6100 has the highest negative effect on the input gene expression signature

Rank	Drug	Dose	Score
CD44+/CD24− primary tumorigenic cells versus rest			
1	Moroxydine	19 μM	1
6	Trichostatin A	100 nM	0.951
7	Idoxuridine	11 μM	0.951
**6039**	**Tretinoin**	**1 μM**	**−0.746**
**6058**	**Pioglitazone**	**10 μM**	**−0.775**
**6100**	**CP-863187**	**10 μM**	**−1**
Normal and metastatic CD44+ *vs* normal and metastatic CD24+ cells			
1	Fulvestrant	1 μM	1
19	Trichostatin A	100 M	0.918
**5910**	**Poiglitazone**	**10 μM**	**−0.728**
**5971**	**Tretinoin**	**1 μM**	**−0.759**
**6100**	**Enalapril**	**8 μM**	**−1**
	Transformed SSEA1+ fibroblasts *vs* SSEA1-fibroblasts		
1	Acenocoumarol	11 μM	1
4	Fulvestrant	1 μM	0.973
**5971**	**Tretinoin**	**13 μM**	**−0.764**
**6100**	**Denatonium benzoate**	**9 μM**	**−1**
MCF10A CD44+/CD24− versus MCF10A CD44−/CD24+			
1	LY-294002	10 μM	1
2	Trichostatin A	100 nM	0.949
**6086**	**Tretinoin**	**1 μM**	**−0.769**
**6098**	**Poiglitazone**	**10 μM**	**−0.923**
**6100**	**11-deoxy-16,16-dimethylprostaglandin E2**	**10 μM**	**−1**
CD271+ basal-like stem cells versus differentiated luminal-like cells			
1	Fusidic acid	7 μM	**1**
2	Captopril	17 μM	0.993
**5948**	**Tretinoin**	**1 μM**	**−0.636**
**6099**	**Acetylsalicylic acid**	**100 μM**	**−0.999**
**6100**	**Ticlopidine**	**13 μM**	**−1**
GD2+ stem cells versus non stem cells			
1	Furazolidone	18 μM	1
2	Cefmetazole	8 μM	0.959
**5899**	**Rosiglitazone**	**10 μM**	**−0.727**
**5997**	**Tretonin**	**1 μM**	**−0.786**
**6100**	**Ioxaglic acid**	**3 μM**	**−1**

**Table 2:** Connectivity map analysis of TNBC subtypes

Rank	Drug	Dose	Score
Basal-like 1 TNBCs versus rest of TNBCs			
1	N-acetylmuramic acid	14 μM	1
2	Paclitaxel	5 μM	0.947
5	Metformin	24 μM	0.916
**6099**	**Oxantel**	**7 μM**	**−0.95**
**6100**	**Azathioprine**	**14 μM**	**−1**
Basal-like 2 TNBCs versus rest of TNBCs			
1	N-acetylmuramic acid	14 μM	1
2	Paclitaxel	5 μM	0.947
5	Metformin	24 μM	0.916
**6099**	**Oxantel**	**7 μM**	**−0.95**
**6100**	**Azathioprine**	**14 μM**	**−1**
Immunomodulatory TNBCs versus rest of TNBCs			
1	Chlorpromazine	11 μM	1
3	Metformin	24 μM	0.968
**6099**	**Morantel**	**11 μM**	**−0.993**
**6100**	**Trichostatin A**	**100 nM**	**−1**
Luminal Androgen Receptor TNBCs versus rest of TNBCs			
1	Trichostatin A	1 **μ**M	1
2	Fulvestrant	1 μM	0.957
**6096**	**Poiglitazone**	**10 μM**	**−0.949**
**6089**	**Rosiglitazone**	**10 μM**	**−0.901**
**6100**	**AR-A014418**	**10 μM**	**−1**
Mesenchymal stem like TNBCs versus rest of TNBCs			
1	Sirolimus	100 nM	1
2	Colforsin	50 μM	0.95
4	Resveratrol	50 μM	0.949
5	Fulvestrant	1 μM	0.943
**6096**	**Rosiglitazone**	**10 μM**	**−0.95**
**6097**	**Tretinoin**	**1 μM**	**−0.962**
Mesenchymal TNBCs versus rest of TNBCs			
1	Allantoin	25 μM	1
2	Chrysin	16 μM	0.971
**6086**	**Tretinoin**	**1 μM**	**−0.841**
**6099**	**Paroxetine**	**1 μM**	**−0.934**
**6100**	**Verteporfin**	**3 μM**	**−1**

## Notes

**Grant Support:** NIH (R21 CA152536-01A1) and Susan G. Komen for the Cure (SAC110025) grants to HN.
