# Supplementary material for: Identification of FDA-approved Drugs Targeting Breast Cancer Stem Cells Along With Biomarkers of Sensitivity
Source: Sci Rep. 2013 Aug 28;3:2530. doi: 10.1038/srep02530 (PMC3965360; doi:10.1038/srep02530)
Supplement: Supplementary Information — Supplementary Table S1 and Figure S1 [file srep02530-s1.pdf]

Supplementary Table 1:

Identification of FDA-approved Drugs Targeting Breast Cancer Stem Cells Along With Biomarkers of Sensitivity

Poornima Bhat- Nakshatri[1], Chirayu P Goswami[2], Sunil Badve[3], George W. Sledge Jr.[4$], and Harikrishna Nakshatri[1,5*]

Departments of Surgery[1], Pathology and Laboratory Medicine[3], Medicine[4], and Biochemistry and Molecular Biology[5], Center for Computational Biology and Bioinformatics[2], Indiana University School of Medicine, Indianapolis, IN 46202, USA

Running title: Therapeutic targeting of breast cancer stem cells

Key words: Breast cancer, cancer stem cells, retinoic acid, SOX2, connectivity map

$ Current Address: Stanford University School of Medicine, Paolo Alto, CA, USA

Primers used for qRT-PCR

CDX2-F: TGG TGT ATG CAC AGG GTG TGG TAT

CDX2-R: AAT GAC AGG AAG TCC AGG TTG GCT

SOX2-F: CAC ATG AAG GAG CAC CCG GAT TAT

SOX2-R: GTT CAT GTG CGC GAA CTG TCC AT

SERPINE1-F: AAT GTG TCA TTT CCG GCT GCT GTG

SERPINE1-R: ACA TCC ATC TTT GTC CCC TAC CCT

SLUG-F: TTT CTG GCC TGG CCA AAC ATA AGC

SLUG-R: ACA CAA GGT AAT GTG TGG GTC CGA

βACTIN-F: AAT GAG GCC GAG GAC TTT GAT TGC

βACTIN-R: AGG ATG GCA AGG GAC TTC CTG TAA

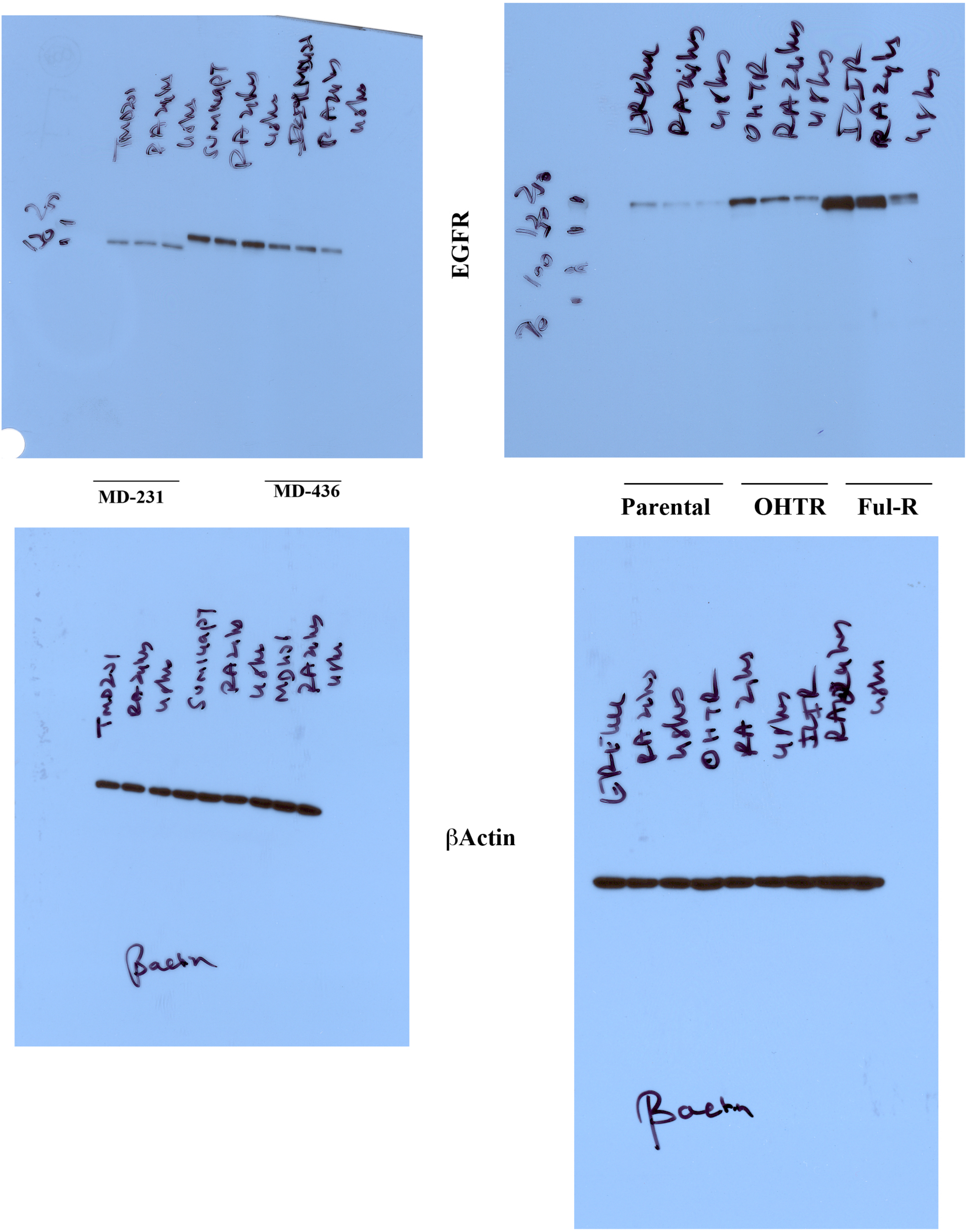

Figure S1: The uncropped immunoblotting data for Figure 3. All samples were obtained under identical experimental condition and all the lanes in one gel data were processed in parallel. Blots immunoblotted for EGFR were reprobed for βActin. Results of SUM149 cells (loaded in between TMD-231 and MD-436 were not used in the manuscript.
